# Liver injury secondary to chest tube placement: a case report of conservative management and review of literature

**DOI:** 10.1002/ccr3.1280

**Published:** 2017-11-22

**Authors:** Badr Serji, Houda Mirali, Mohammed Chablou, Imane Kamaoui, Tijani El Harroudi

**Affiliations:** ^1^ Surgical Oncologic Department Faculty of Medicine and Pharmacy Mohammed Ist University Oujda Morocco; ^2^ Department of Radiology Mohammed VI^th^ Hospital Faculty of Medicine and Pharmacy Mohammed Ist University Oujda Morocco

**Keywords:** Chest tube, complications, conservative management, liver injury

## Abstract

Chest tube placement is a routine procedure performed in different medical departments. Liver injury is a very rare complication that can occur but can be life‐threatening. Conservative management can be proposed in stable patient. Following guidelines and training physicians should decrease the incidence of such complications.

## Introduction

Chest tube placement is an invasive procedure that is commonly used by different specialty physicians 1. In the emergency department, the main indication is pneumothorax secondary to thoracic injury 2. We report a case of hepatic injury secondary to chest tube placement treated with the successful conservative method.

## Case Report

**Figure 1:**
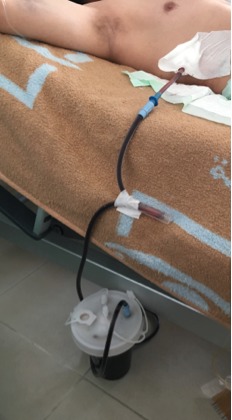
Image showing the low position of chest tube.

**Figure 2:**
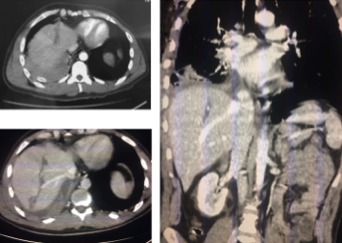
Images of abdominal CT showing the path of the chest tube thru the liver next to hepatic right and middle hepatic vein.

**Figure 3:**
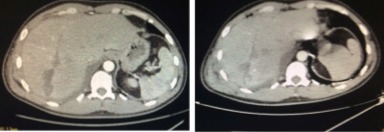
Abdominal CT at day 7 after ablation showing the cicatrization of the liver and resorption of initial perihepatic hemoperitoneum.

We report the case of a 42‐year‐old man, with no past medical history, who was admitted to an emergency department at a peripheral healthcare center for a penetrating thoracic injury secondary to aggression using a knife. At admission, patient had dyspnea with an open blowing wound localized in the right parasternal side at the fourth intercostal space. After patient conditioning and monitoring, he had a standard chest radio that showed a right pneumothorax. The medical team decided to place a chest tube thoracostomy. Under local anesthesia, a senior emergency doctor put the chest tube in place. Unfortunately, the drain did not bring air but only massive bleeding. Physicians concluded to a massive hemopneumothrax, but the patient had palpitation with coldness and weakness. The drain was clamped immediately and the patient transfused with two red blood cells unit. After stabilization, he was transferred to our tertiary center for massive hydropneumothorax. The patient arrived with a mild dyspnea, blood pressure was 90/65 mmHg, and pulse was 95/min, saturation after monitoring was at 94% and 98% under oxygen therapy. The examination showed a sutured injury in right parasternal side. The patient had a right thoracic chest tube clamped and placed in the seventh intercostal space (Fig. 1). The patient complained of a discrete sensitivity at palpation of the right hypochondrium. We suspected a liver injury secondary to chest tube placement. Biology showed hemoglobin level at 10.2 g/dL, white blood cells at 20,120/mm^3^, platelet at 190,000/mm^3^, CRP = 233.35 mg/L, ASAT = 66 UI/L, ALAT = 132 UI/L, GGT = 177 UI/L, and ALP = 162 UI/L. Abdominal CT scan confirmed the diagnosis of liver trauma secondary to a misplaced thoracostomy tube, with a mild hemoperitoneum localized to the liver area. The drain path in the liver was high avoiding hilum structure and fortunately avoiding right and middle hepatic vein (Fig. 2). CT scan showed a mild hemothorax that does not need chest drainage after consultation with the thoracic surgeon. Considering the stable hemodynamic situation of the patient, we decided to be conservative and to remove the drain gradually by 2 cm each day (while keeping it clamped) under strict monitoring. At day 4, the drain was totally removed. Control showed hemoglobin level at 8.4 g/dL. The patient was transfused with two red blood cells unit, and control was at 10.6 g/dL. A CT scan control at day 7 showed no hemoperitoneum (Fig. 3), but we could see the path of the drain into the liver without active bleeding and the patient did not report any digestive bleeding and transit was normal. There was a persistence of atelectasis in the right lung, so the patient was transferred in thoracic surgery to manage it.

## Discussion

Pleural disease requiring placement of chest tube is frequent and multiple 3. It can concern traumatic situation 4, malignant effusion 5, or postoperative management 6. In the emergency department, more than 50% of patients presenting a thoracic injury require a thoracostomy 7. Complications can occur during the procedure or during chest tube maintenance with a range from 5% to 10% and may concern lung, heart, intercostal pedicle, diaphragm, spleen, and bowel and the list is not exhaustive 3. Hepatic injury secondary to chest tube insertion is very rare, and its management is not standardized 8, 9, 10. Liver lesions can very rarely be life‐threatening and may require emergency surgery 11. In our case, the conservative strategy was preferred because of the stable hemodynamic situation of the patient and to avoid invasive surgery as possible. The study of the CT scan with radiologists showed that intrahepatic biliary and portal structures were safe and the right and middle hepatic veins were close but seem to be intact. Some authors reported successful conservative ablation of a chest tube in the liver using embolization technique 9, 10 or simply by removing the drain under close monitoring 8. Our strategy was to retrieve the drain gradually each day, while keeping it clamped, to ensure progressive parenchymal hemostasis in the released part so the drain plays the role of a piston, which blocks the spread of the hemorrhage if it occurs. The success of a conservative strategy can be controlled immediately by fluoroscopy when embolization is used 9, 10 or by CT scan 8. Our patient had no further complications after drain removal and control was made using a CT scan. If we resume literature, there are only five published cases of liver injury secondary to thoracic drainage (Table 1) 8, 9, 10, 11, 12. Only one required an open surgery to remove the drain and stop hemorrhage in an unstable patient 11. Three studies report a conservative management. Two of them used embolization technique by coils to remove the drain 9, 10, and in the other one, authors did not use any particular method because they estimate that the path of the drain was blinded at its end 8. In the last case, authors did not specify their methodology of drain ablation, but they precise that it was done in the operating room 12. Such incident raises again the question of teaching physicians, in different specialty (and especially in emergency departments), a safe procedure to put a chest tube and their ability to follow guidelines 13, 14. A recent survey showed that incident after chest tube placement is not totally reported 15. Moreover, it showed that it is not only caused by trainers but also by senior doctors 15. Another study published previously found that there was a flaw in teaching young doctors 16. Forty‐five percent of questioned doctors in this study localized the area of chest tube placement outside the triangle of safety and 73% of doctors with no experience in chest tube placement did too 16.

**Table 1:** Table resuming cases of liver injury secondary to chest tube placement reported in literature

	Age	Gender	Indication of chest tube	Hemodynamic	Treatment
Sommacale et al.	44	M	Pleural effusion (Pneumopathy)	Stable	Conservative
Tait et al.	82	F	Pneumothorax	Stable	Conservative (Embolization: coils)
Hegarty et al.	26	F	Not precise	Stable	Conservative (Embolization: coils)
Bae	72	F	Pleural effusion	Unstable	Surgery
Gorospe et al.	76	F	Pleural effusion (Lung cancer)	Stable	Not precise

M, Male; F, Female.

Liver trauma after chest tube placement is very rare and can be managed conservatively in a stable patient. Teaching physicians safe procedures is the best way to avoid such incident.

## Authorship

BS: reviewed the literature, wrote the manuscript, and is healthcare provider. MC: collected data. HM: collected data and revised the English version of the manuscript. IK: revised CT images and interpretation. TEH: revised the manuscript.

## Conflict of Interest

Authors have no conflict of interests or financial support to disclose.

## References

[1] Gilbert, T. B. , B. J. McGrath , and M. Soberman . 1993 Chest tubes: indications, placement, management, and complications. J. Intens. Care Med. 8:73–86. 10.1177/088506669300800203 10148363

[2] Bailey, R. C. 2000 Complications of tube thoracostomy in trauma. J. Accid. Emerg. Med. 17:111–114. 10718232 10.1136/emj.17.2.111 PMC1725338

[3] Mao, M. , R. Hughes , T. J. Papadimos , and S. P. Stawicki . 2015 Complications of chest tubes: a focused clinical synopsis. Curr. Opin Pulm. Med. 21:376–386. 26016583 10.1097/MCP.0000000000000169

[4] Schmidt, U. , M. Stalp , T. Gerich , M. Blauth , K. I. Maull , and H. Tscherne . 1998 Chest tube decompression of blunt chest injuries by physicians in the field: effectiveness and complications. J. Trauma 44:98–101. 9464755 10.1097/00005373-199801000-00010

[5] van den Toorn, L. M. , E. Schaap , V. F. Surmont , E. M. Pouw , K. C. van der Rijt , and R. J. van Klaveren . 2005 Management of recurrent malignant pleural effusions with a chronic indwelling pleural catheter. Lung Cancer 50: 123–127. 15998551 10.1016/j.lungcan.2005.05.016

[6] Kejriwal, N. K. , and M. A. Newman . 2005 Use of a single silastic chest drain following thoracotomy: initial evaluation. ANZ J. Surg. 75:710–712. 16076338 10.1111/j.1445-2197.2005.03479.x

[7] Chrysou, K. , G. Halat , B. Hoksch , R. A. Schmid , and G. J. Kocher . 2017 Lessons from a large trauma center: impact of blunt chest trauma in polytrauma patients‐still a relevant problem? Scand J. Trauma Resusc. Emerg. Med. 25:42. 28427480 10.1186/s13049-017-0384-y PMC5399315

[8] Sommacale, D. , M. Lhuaire , T. Piardi , E. Palladino , and R. Kianmanesh . 2013 Chest drain in the liver. Liver Int. 33:958. 23410130 10.1111/liv.12116

[9] Tait, P. , U. Waheed , and S. Bell . 2009 Successful removal of malpositioned chest drain within the liver by embolization of the transhepatic track. Cardiovasc. Intervent. Radiol. 32:825–827. 18972157 10.1007/s00270-008-9461-y

[10] Hegarty, C. , J. F. Gerstenmaier , and D. Brophy . 2012 Chest tube’ removal after liver transgression. J. Vasc. Interv. Radiol. 23:275. 22264554 10.1016/j.jvir.2011.10.010

[11] Bae, J. M. 2015 Life threatening hemoperitoneum and liver injury as a result of chest tube thoracostomy. Clin. Med. Insights Case Rep. 8:15–17. 25780345 10.4137/CCRep.S23139 PMC4345926

[12] Gorospe, L. , G. M. Muñoz‐Molina , and A. P. Valdebenito‐Montecino . 2016 A malpositioned chest tube within the liver. Asian Cardiovasc. Thorac. Ann. 24:612. 25834127 10.1177/0218492315579556

[13] Laws, D. , E. Neville , and J. Duffy . 2003 BTS guidelines for the insertion of a chest drain. Thorax 58(suppl II):55–59. 10.1136/thorax.58.suppl_2.ii53 PMC1766017 12728150

[14] Henry, M. , T. Arnold , and J. Harvey . 2003 BTS guidelines for the management of spontaneous pneumothorax. Thorax 58(suppl II):ii39–ii52. 12728149 10.1136/thorax.58.suppl_2.ii39 PMC1766020

[15] Harris, A. , B. R. O'Driscoll , and P. M. Turkington . 2010 Survey of major complications of intercostal chest drain insertion in the UK. Postgrad. Med. J. 86:68–72. 20145053 10.1136/pgmj.2009.087759

[16] Griffiths, J. R. , and N. Roberts . 2005 Do junior doctors know where to insert chest drains safely? Postgrad. Med. J. 81:456–458. 15998822 10.1136/pgmj.2004.024752 PMC1743306

